# News and Notes

**Published:** 1905-12

**Authors:** 


### *NEWS AND NOTES.*

---

DR. J. D. CROMER, has been in New York.

---

DR. T. J. CHARLTON, of Savannah, is in New York under the care of Dr. Wylie.

---

DR. W. B. CRAWFORD, of Savannah, is suffering from an attack of typhoid fever.

---

DR. M. D. WELLBORN, Ezell Station, Ala., was fined $100 and cost for selling cocaine.

---

DR. CHARLES R. ANDREWS and Miss May Waldo, both of Atlanta, were married November 8.

---

DR. S. M. DEAL, of Columbia, S. C., recently spent a week in Atlanta visiting his sister, Mrs. J. S. Scott.

---

DR. W. A. SELMAN, of Atlanta, and Miss Elizabeth Crouch were married in Gay, Ga., Nov. 15th.

---

THE University of Giessen will celebrate the three-hundredth centenary of its foundation in May, 1907.

---

DR. W. A. TURNER, of Newnan, Ga., and Miss Annie K. Dowdell, of Opelika, Ala., were married October 18.

---

DR. V. C. VAUGHN, of Ann Arbor, Michigan, delivered the opening lecture of the Medical Faculty of Toronto University on October 3d.

DR. W. H. WILDER was elected president of the Tri-State Medical Association of Tennessee, Georgia and Alabama.

---

DR. THOMAS J. COLLIER, of Atlanta, Ga., and Miss Gertrude McCarley, of Chattanooga, Tenn., were married Oct. 10th.

---

THE degree of Doctor of Science was recently conferred on Dr. John B. Murphy, of Chicago, by the University of Illinois.

---

HOT SPRINGS, ARK., has been selected as the place for the next annual meeting of the Mississippi Valley Medical Association.

---

DR. W. C. WILLIAMS, colored, was fined $25 and costs, September 18, for practicing medicine in Atlanta without a license.

---

A MOVEMENT is on foot in Washington, D. C., to secure an appropriation of $150,000 from Congress for a municipal hospital.

---

THE Emperor of Austria has conferred a life patent of nobility on Dr. Edmund Neusser, professor of medicine in the University of Vienna.

---

THE Secretary of War has issued an order establishing a department of military hygiene at the U. S. Military Academy at West Point.

---

DR. EDWARD C. REGISTER, editor of the *Charlotte Medical Journal,* has been elected president of the North Carolina Medical Society.

---

DR. J. FRANK HUSS, of Atlanta, Ga., and Miss Annie Lamar Jackson, were married at the home of the bride, Covington, Ga., Oct. 25, 05.

DR. JAMES H. ATLEE, of Chattanooga, recently spent several days in Atlanta. Dr. Atlee is associate editor of *Southern Medicine and Surgery.*

---

THE number of deaths from tuberculosis in England is 60,000 a year; yet it is said there are only seventy sanatoriums, with room for 2,760 patients.

---

DR. LUTHER CHILES, of Norfolk, Va., charged with performing a criminal abortion on Miss Sarah Atkinson, has been released on bail of $10,000.

---

DR. EDWARD MARTIN has resigned his position as director of the Department of Health and Charities of Philadelphia. The position paid $10,000 a year.

---

IT is announced that a hospital for epileptics at Moscow, Russia, is to be built at once, with immediate accommodations for 150 patients, and possibility of its enlargement.

---

JOHN SLAVENS, M.D., University of Louisville (Ky.) Medical Department, 1848, an old resident of Putnam county, Indiana, died at his home in Brick Chapel, October 10th, aged 94.

---

THE doctors of Meriwether county have organized a County Medical Association, with Dr. E. B. Terrell, president, Dr. Snead, vice-president, and Dr. P. W. Fitts, secretary and treasurer.

---

THE Augusta board of health has sent out personal letters to the physicians of the city urging on them the necessity of full and prompt reports of all communicable diseases and of births and deaths.

---

JOHN W. OGILVIE, M.D., a graduate of the Medical College of the State of South Carolina, Charleston, 1845, died at his home near Allendale, S. C., September 27th, after a lingering illness, aged 87.

---

CARLSBAD recently celebrated the fiftieth annual visit of a Moravian manufacturer. Much ceremony attended his return to the springs this year and the mayor announced that one of the walks is to be named after him.

---

IT is reported that a bill will be submitted to the Legislature of Massachusetts at its coming session "to prohibit all experiments under any circumstances and for any purpose whatsoever, with or without anesthetics, upon dogs and cats."

---

SWIFT-JONES WEDDING.—Miss Lena Swift and Dr. Willis Jones were married October 25, at the home of the bride's mother, Mrs. J. P. Huntley. Several hundred guests were present at the ceremony and the reception which followed.

---

THE French Parliament will offer a $200,000 prize for a cure of tuberculosis. It is expected that the prize will stimulate research in time for the result to be announced at the next tuberculosis congress, which is to be held in Washington.

---

PROFESSOR CHANTEMESSE, a member of the Pasteur Institute, lecturing at the Academy of Medicine last week, said his experiments had proved that flies were the greatest disseminators of cholera in the towns.—*Med. News, October 28th.*

---

A 100-MILE ride to patients was taken by Dr. Richards from his home, Thermopolis, to Kerwin, Wyoming, where several men were killed by an explosion in a gold mine. The mountainous distance was covered in eleven hours. Four relays were used, the ranchmen along the route supplying the horses.

THE Muskogee County Medical Society, at its regular meeting, October 4th, adopted a resolution requesting the newspapers of the city not to publish the names of attending physicians in their accounts of accidents. The object of the resolution is to prevent even the semblance of advertising on the part of the physician.

---

THE names of twelve Frankfort physicians have been presented to the prison commissioners, each of whom agrees to serve as prison physician for a month without pay, or until the expiration of the term of office of the late Dr. Hugh L. Tobin, provided the salary is given to the widow of the deceased physician.—*Jour. A. M. A.*

---

DR. C. M. STANLEY died at his home on Bayou Barataria, La., October 9. Dr. Stanley came to Louisiana from North Dakota three years ago and at the outbreak of yellow fever devoted much of his time to the sick. On September 17 he was attacked with yellow fever, but recovered of this and unfortunately went back to his work too early, which resulted in a relapse and his death.

---

IT is announced by the publishers of the *New York Medical Journal* that arrangements have been made for a consolidation of the above journal and *Medical News,* which has been published since 1843 by the well-known house of Lea Brothers & Co. This union will no doubt furnish us with one of the best weekly journals published in this country.

---

IT is reported that Dr. Zachary T. Young of St. Laundry, La., was shot and killed by Dr. T. Thompson. There had been trouble between the physicians for some time and October 5th, while out driving, both met on the highway and each refused to make room for the other to pass. They immediately drew weapons and Thompson firing first killed Young. Dr. Young was a prominent physician of St Laundry Parish.—*St. Louis Med. Review.*

THE registration just closing in the Cornell University Medical College shows a decrease over the best of the previous years. The three advanced classes are somewhat larger than usual, but the first-year class, which hitherto has contained an average of about 130 students, this year has less than 120. Medicine at present does not seem as attractive a calling as business, although eighty-five per cent. of the graduating class last year obtained hospital positions, and the hospital graduate has undoubtedly a better chance of success than others.—*Jour. A. M. A.*

---

ESTABLISHMENT of a permanent medical home and library for the doctors of Chicago was discussed at a dinner of the Physicians' Club at the Sherman House on October 24th. It is proposed to spend $2,000,000 and to raise $500,000 to start work. Dr. Daniel R. Brower suggested that 100 physicians give $5,000 each to start the movement. Dr. Nicholas Senn was the first to pledge that sum. A committee of the Chicago Medical Society now has the work in hand. Members of all organizations made up entirely of physicians and surgeons have given the project their hearty approval and promised funds to carry it out.—*N. Y. and Phila. Med. Jour. Nov. 4th.*

---

THE committee in charge of the International Medical Congress, which will be held in Lisbon from April 19 to 26, 1906, has written asking for the contribution of papers on the following medico-legal subjects, and saying that as yet no titles of communications touching on any of these subjects have been received from this country:

The Signs of Virginity and of Defloration in Medico-Legal Relations.

Hand Marks and Finger Prints; Their Medico-Legal Importance.

The Medico-Legal Importance of the Carunculæ Myrtiformes.

The Mechanism of Death by Hanging.

The Value of Bacteriologic Examination of Vulvo-Vaginal Discharges in the Determination of Venereal Contagion.

The Signs of Death by Drowning.

Ecchymoses in Legal Medicine.

Spontaneous and Criminal Abortions from a Medico-Legal Point of View.

Medico-Legal Investigations of Blood Stains.

The Relations between the Seat of Cerebral Contusions and the Point of Application of the Agent which Produced Them.

Epilepsy in Legal Medicine.

The Induction of Abortion; When Is It Permissible?

The Value of Legal Medicine in the Study of Criminal Law.

The Best Legislation for the Protection of the "Medical Secret" (the obligation imposed upon physicians to treat as inviolable all information concerning patients obtained while in the discharge of their professional duties).

The Effects of the Civil and Penal Law Towards the New-Born Living Infant.

Distinction Between the Natural Openings in the Hymen and Tears in this Membrane.

Criminal Vulvar Copulation.

Organization of Medico-Legal Services.

If any of the readers of this communication intend to take part in the discussions of this section of the congress, or to prepare papers for it on any of the subjects mentioned, or on any other subject in medicine or surgery, he should inform the Secretary of the American Committee.

RAMON GUITERAS,
Secretary, American National Committee,
75 West Fifty-fifth Street, New York.

---

### INTERNATIONAL MEDICAL CONGRESS.

I am pleased to announce that final arrangements have been perfected for the tour of the American party to the International Medical Congress at Lisbon, April, 1906.

The party will sail on Saturday, April 7, on the North German Lloyd steamer, "Koenig Albert" for Gibraltar, visiting Algerciras, Seville, Cordova, etc., spend a week in Lisbon during the Congress and returning to New York on Wednesday, May 9. This trip may be made comfortably in a first-class steamer both ways, all expenses paid, including board and lodging while in Lisbon, and entertainment at other points, for $300.00.

A number of side trips are added and ticket will be good returning through Europe, if desired, at a slightly increased cost.

Following is a list of those who have joined the party:

Lewis S. McMurtry, M.D., Louisville.
Nicholas Senn, M.D., Chicago.
J. D. Griffith, M.D., Kansas City, Mo.
W. F. Southard, M.D., San Francisco.
Frank P. Norbury, M.D., Jacksonville, Ill.
W. T. Corlett, M.D., Cleveland, O.
C. H. Hughes, M.D. St. Louis, Mo.
R. T. Morris, M.D., New York City.
A. Vander Veer, M.D., Albany, N. Y.
Jos. M. Mathews, M.D., Louisville.
J. B. Murphy, M.D., Chicago.
Fenton B. Turck, Chicago.
Jas. E. Moore, M.D., Minneapolis, Minn.
Ramon Guiteras, New York City.

Dr. John H. Musser (Philadelphia) is chairman of the National American Committee, and Dr. Ramon Guiteras (75 West Fifty-fifth street, New York City) is the secretary, to whom all applications for membership and communications in regard to the presentation of papers should be addressed.

All those who contemplate the trip are cordially urged to make reservation with the writer at once in order to secure desirable berth on the steamer and good hotel accommodations. Program of the itinerary upon request.

CHAS. WOOD FASSETT, St. Joseph, Mo.

